# *INF-γ*基因转染肺泡巨噬细胞抗肿瘤活性的研究

**DOI:** 10.3779/j.issn.1009-3419.2011.05.13

**Published:** 2011-05-20

**Authors:** 凤丽 周, 筱刚 毕, 天托 张, 静 黄

**Affiliations:** 510630 广州，中山大学附属第三医院呼吸科 Department of Respiratory Medicine, the Tird Afliated Hospital, Zhongshan University, Guangzhou 510630, China

**Keywords:** 肺肿瘤, 肺泡巨噬细胞, 基因转染, Lung neoplasms, Alveolar macrophage, Gene transferction

## Abstract

**背景与目的:**

活化的肺泡巨噬细胞（alveolar macrophage, AM）具有抗肿瘤功能，γ干扰素（interferon-γ, INF-γ）是巨噬细胞的活化因子之一，其与巨噬细胞体外共同培养可增强巨噬细胞的免疫功能。本研究旨在了解人*INF-γ*基因体外转染肺癌患者的AM后对其抗肿瘤功能的影响。

**方法:**

经肺泡灌洗获AM，分离纯化，以*INF-γ*基因转染AM，以RT-PCR方法和ELISA方法检测人*INF-γ*基因的成功转染；分别检测AM产生TNF-α、NO、IL-1的水平及AM杀伤L1210细胞的活性。

**结果:**

RT-PCR方法和ELISA方法均显示人*INF-γ*基因已成功转染AM；经人*INF-γ*基因转染后，肺癌患者AM产生TNF-α、NO、IL-1的水平较对照组明显升高（*P* < 0.05）；AM杀伤L1210细胞的活性较对照组明显增强（*P* < 0.05）。

**结论:**

*INF-γ*基因体外转染肺癌患者的AM，能使AM的抗肿瘤活性明显增强。

## Abstract (en)

**Background and objective:**

Alveolar macrophages (AMs) activated have the antitumor activity. The interferon-γ (INF-γ) is one of the stimulating factors. INF-γ can enhance the immune function of AMs *in vitro*. The aim of this study is to investigate the effect of human *INF-γ* gene on the antitumor activity of AMs when transfected into the alveolar macrophages (AMs) from the patients with lung cancer *in vitro*.

**Methods:**

AMs obtained by brochoalveolar lavage were separated and transfected by *INF-γ* gene. RT-PCR and ELISA were applied to determine whether the transfection was successful. The levels of tumor necrosis factor α (TNF-α), nitric oxide (NO) and interleukin-1 (IL-1) produced by AMs and the killing activity of AMs against L1210 cells was detected respectively.

**Results:**

Both RT-PCR and ELISA demonstrated that human *INF-γ* gene had been successfully transfected into AMs. When transfected by human *INF-γ* gene, the levels of TNF-α, NO and IL-1 produced by AMs from the patients with lung cancer and the killing activity of AMs against L1210 cells were significantly higher than those of the control groups.

**Conclusion:**

Human *INF-γ* gene can enhance the antitumor activity of AMs when transfected into AMs from the patients with lung cancer.

肺癌的发病率和死亡率呈逐年上升的趋势，已经成为一种严重威胁人类健康与生命安全的疾病。目前肺癌的治疗疗效仍然未取得突破性的进展，寻找新的多方面的治疗途径仍然是目前需要解决的问题，免疫基因治疗就是有待研究的主要治疗途径之一。

肺泡巨噬细胞（alveolar macrophage, AM）是肺部防御的首要细胞，占正常人支气管肺泡灌洗液中细胞总数的80%以上。AM具有多种抗肿瘤功能，能通过直接融解和吞噬作用，还能分泌多种细胞因子，如：肿瘤坏死因子α（tumor necrosis factor α, TNF-α）、一氧化氮（nitric oxide, NO）、白细胞介素I（interleukin I, IL-1）等，起到抗肿瘤的作用。活化的AM抗肿瘤活性明显增强，干扰素-γ（interferon-γ, INF-γ）是很强的巨噬细胞活化因子，直接作用于巨噬细胞即能促进巨噬细胞的吞噬和分泌功能^[1]^。利用基因转染技术将某些特殊的基因转入巨噬细胞从而改变巨噬细胞的免疫功能的研究国内外均有报道^[2-5]^，但多为动物（鼠）的腹腔AM的实验或人AM的非抗肿瘤功能的研究。本文设计利用INF-γ体外转入肺癌患者AM，观察AM抗肿瘤功能的变化，为临床应用基因转染技术于肺癌的免疫基因治疗提供理论依据。

## 1 材料与方法

### 1.1 实验对象

肺癌患者，共30例，均经病理证实，其中小细胞肺癌8例，非小细胞肺癌22例。既往未经任何治疗。

### 1.2 AM的收集

按常规纤维支气管镜操作进行支气管肺泡灌洗（bronchoalveolar lavage, BAL），以37 ℃的灭菌生理盐水，每次50 mL，注入后立即抽吸，反复4次，收集灌洗液。以贴壁分离的方法分离和纯化AM，台盼蓝染色，倒置显微镜下鉴定。

### 1.3 将人INF-γ CDNA构建于真核表达质粒，获表达人*INF-γ*基因的真核表达载体

利用双酶切定向克隆技术，将人INF-γ全长cDNA与表达质粒pREP-8的DNA相连接，构建成真核表达质粒Prep-8-INF-γ（由上海锐谷生物科技有限公司完成）。

### 1.4 将纯化的AM与人*IFN-γ*基因转染液共同孵化

在24孔板上分别加入AM 1×10^6^/孔，*INF-γ*基因转染液100 μL/孔（设不表达INF-γ的质粒对照和磷酸钙对照）。置37 ℃、5%CO_2_中孵育2 h，洗去转染液。孵育后的AM，加含10%小牛血清的RPMI-1640完全培养基继续培养48 h。收集培养上清液及AM。

### 1.5 RT-PCR方法检测转染了*INF-γ*基因的AM总RNA的RTPCR产物

### 1.6 上清液检测

#### 1.6.1 INF-γ的活性测定

采用ELISA法^[6]^。

#### 1.6.2 TNF-α含量测定

采用ELISA法。按北京邦定生物医学公司试剂盒提供的操作程序进行，测波长570 nm处的OD值。

#### 1.6.3 NO含量测定

采用Gries法。将亚硫酸钠标准液稀释为320 μmol/L、160 μmol/L、80 μmol/L、40 μmol/L、20 μmol/L、10 μmol/L、和5 μmol/L 7个浓度，加入96孔板，每份样品3个复孔；将待测AM培养上清和空白对照（只加蒸溜水）加入另一行96孔板中，所有样品均加0.1 mL，均为3个复孔；然后各孔加入Gries试剂0.1 mL，室温显色10 min后用酶标仪上570 nm波长测OD值。用计算和绘制标准曲线即可推算出NO含量。

#### 1.6.4 IL-1含量测定

参照文献^[7]^，样本作1:5稀释，各板设标准品校正孔，依据标准曲线换算各样本中IL-1含量。

### 1.7 AM杀伤活性的检测

用MTT间接比色法^[6]^。收集对数生长期的L1210细胞，经Hanks’液洗后，用RPMI-1640液完全培养基配成1×10^6^/L备用。取1×10^7^/L的AM加入96孔板中，每孔100 μL，1.5 h后以Hanks’液洗2次，再加入L1210细胞（100 μL/孔），每一样品设3个复孔及L1210细胞对照。于37 ℃下24 h后，振荡悬浮细胞。每孔取100 μL加入另一96孔板中，各加入5 g/L的MTT 20 μL/孔，培养4 h后，再加入100 g/L的SDS100 μL/孔，6 h-8 h后，用酶标仪测定570 nm的OD值。并计算杀伤活性。

$
					杀伤活性 = \left( {1-\frac{\rm{AM作用后的L1210细胞的OD值}}{\rm{对照L1210细胞的OD值}}} \right) \times 100\% 
					$

### 1.8 统计学方法

使用SPSS 13.0进行统计学分析，实验结果以Mean±SD表示。组间比较采用*t*检验，以*P* < 0.05为差异有统计学意义。

## 2 结果

### 2.1 AM活力测定

对贴壁分离的AM用台盼篮染色法测定细胞活力，其中活细胞不染色，结果表明活细胞率达95%以上。

### 2.2 RT-PCR检测结果

*INF-γ*基因只在转染了Prep-8-INF-γ的AM内转录，在相当于350 bp处有一清晰的条带，而在未转染Prep-8-INF-γ肺癌患者的AM内无转录（无相应的条带），磷酸钙对照亦无相应的条带出现（图 1）。

**1:**
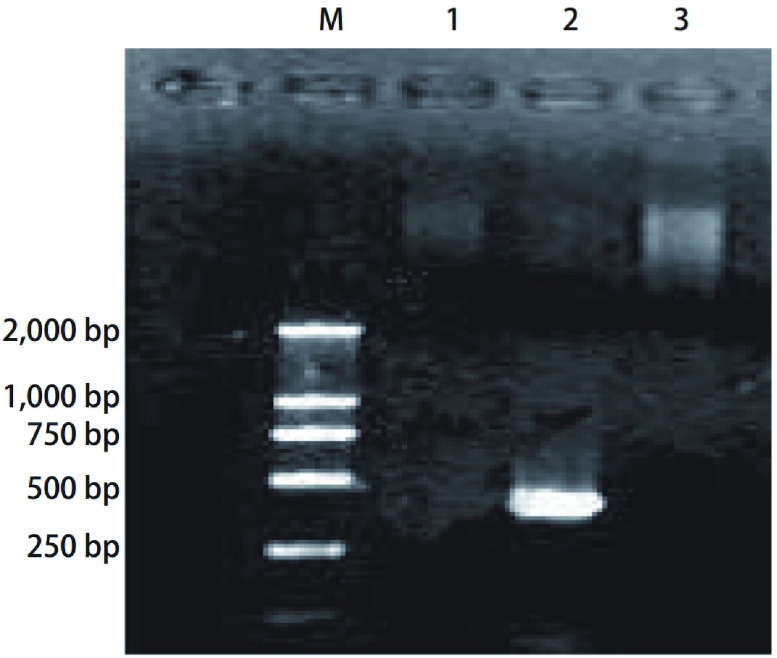
RT-PCR检测AM内*INF-γ*基因转录条带图。M：marker；1：未转染*INF-γ*基因的肺泡巨噬细胞中总RNA的RT-PCR产物；2：转染了*INF-γ* 基因的AM总RNA的RT-PCR产物；3：磷酸钙对照 the figure of *INF-γ* gene transcription of AMs with RT-PCR. M:marker; 1: the RT-PCR product of total RNA in AMs that *INF-γ* gene were not transfected into; 2: the RT-PCR product of total RNA in AMs that *INF-γ* gene were transfected into; 3: the control of calcium phosphate

### 2.3 *INF-γ*基因转染后巨噬细胞培养上清液中INF-γ活性的检测结果

*INF-γ*基因转染AM培养上清液中4 h即能检测到INF-γ，7 d后检测到INF-γ的水平为（34.5±3.2）ng/mL；而不表达INF-γ的质粒对照组检测到的INF-γ平均值为（5.5±1.6）ng/mL。基因转染组和对照组比较INF-γ水平，差异有统计学意义（*t*=13.739, *P* < 0.001）。

### 2.4 *INF-γ*基因转染后巨噬细胞培养上清液中TNF-α、NO和IL-1水平的检测结果

*INF-γ*基因转染后AM培养上清液中TNF-α水平明显高于质粒对照组，差异有统计学意义（*P* < 0.001）；*INF-γ*基因转染后AM培养上清液中NO水平明显高于质粒对照组，差异有统计学意义（*P* < 0.001）；*INF-γ*基因转染后AM培养上清液中IL-1水平明显高于质粒对照组，差异有统计学意义（*P* < 0.001）（表 1）。

**1:** 基因转染组和对照组AM培养上清液中TNF-α、NO和IL-1水平（Mean±SD） The content of TNF-α, NO and IL-1 in incubating solution of alveolar macrophages of two groups (Mean±SD)

Group	*n*	TNF-*a* (ng/L)	NO (*μ*mol/L)	IL-1 (ng/mL)
INF-*γ* gene were transfected	30	43.2±3.5	90.6±5.9	2, 643±236
Contrast of plasmid	30	20.8±3.2	45.2±4.7	1, 239±198
*t*		15.339	61.673	44.357
*P*		< 0.001	< 0.001	< 0.001

### 2.5 *INF-γ*基因转染后AM对L1210细胞的杀伤活性的检测结果

*INF-γ*基因转染后AM对L1210细胞的杀伤率为（68.9±5.9）%，而质粒对照组对L1210细胞的杀伤率为（15.5±2.1）%，差异有统计学意义（*t*=72.482, *P* < 0.001）。

## 3 讨论

巨噬细胞具有免疫功能，国内外的研究^[2-5]^显示，利用体外基因转染技术可以改变巨噬细胞的免疫活性。抗肿瘤免疫功能是巨噬细胞的重要免疫功能之一，巨噬细胞也是主要的肿瘤免疫细胞，而肺组织中大量的AM起着重要的抗肺癌的免疫功能，巨噬细胞的活化是其抗肿瘤功能的基础，能否通过体外基因转染技术，将巨噬细胞活化因子*INF-γ*基因转入AM，从而使AM活化，使其抗肺癌活性增强，是本研究的目的。

巨噬细胞产生细胞因子是其抗肿瘤活性的主要途径，TNF-α、NO、IL-1是AM分泌的具有抗肿瘤效应的主要细胞因子，TNF-α可引起肿瘤细胞坏死和导致肿瘤细胞凋亡^[8-10]^；IL-1可通过激活细胞毒T淋巴细胞起到杀肿瘤细胞的作用；NO可阻断肿瘤细胞的能量代谢和DNA复制而抑制肿瘤细胞生长，还可诱导肿瘤细胞凋亡^[11]^。本研究结果显示，肺癌患者的AM经体外转染人*INF-γ*基因后，其培养上清液中TNF-α、NO、IL-1的含量均较对照组明显增高（均为*P* < 0.001），提示人*INF-γ*基因转染后，肺癌患者AM产生以上3种细胞因子的活性明显增强，提示其抗肿瘤活性明显增强。

巨噬细胞还可通过吞噬作用和依赖跨膜型肿瘤坏死因子的接触溶解起杀伤肿瘤细胞的作用。小鼠淋巴细胞白血病细胞株L1210细胞被广泛用于巨噬细胞杀瘤活性的检测^[12, 13]^，本研究检测AM杀伤L1210细胞的活性结果显示，转染了人*INF-γ*基因的肺癌患者AM杀伤L1210细胞的能力较对照组明显增强，直接说明肺癌患者AM抗肿瘤活性在转染了人*INF-γ*基因后明显增强。

激活的巨噬细胞才能具有分泌功能和杀伤活性。INF-γ是巨噬细胞激活剂中作用最强的细胞因子之一，INF-γ直接与AM培养都能激活AM使之抗肿瘤活性增强^[1]^。我们采用体外基因转染技术将人*INF-γ*基因转入肺癌患者AM，RT-PCR显示*INF-γ*基因只在转染了Prep-8-INF-γ的AM内转录，同时检测到AM培养上清液中INF-γ的高浓度表达，说明转染是成功的；同时检测到转染了人INF-γ基因的AM培养上清液中TNF-α、NO、IL-1三种细胞因子均较对照组明显升高（均为*P* < 0.001），且其杀伤L1210细胞活性亦明显较对照组增强（*P* < 0.001），提示AM已明显被激活，具有抑瘤和杀瘤的作用。

本研究采用的体外基因转染技术将人*INF-γ*基因转入肺癌患者AM，使其抗肿瘤活性明显增强，为将来临床应用体内基因转染技术增强AM抗肺癌作用的研究提供了实验基础。
